# Development of an Atmospheric Pressure Plasma Jet Device Using Four-Bore Tubing and Its Applications of In-Liquid Material Decomposition and Solution Plasma Polymerization

**DOI:** 10.3390/polym14224917

**Published:** 2022-11-14

**Authors:** Gyu Tae Bae, Hyo Jun Jang, Eun Young Jung, Ye Rin Lee, Choon-Sang Park, Jae Young Kim, Heung-Sik Tae

**Affiliations:** 1 School of Electronic and Electrical Engineering, College of IT Engineering, Kyungpook National University, Daegu 41566, Republic of Korea; 2 The Institute of Electronic Technology, College of IT Engineering, Kyungpook National University, Daegu 41566, Republic of Korea; 3 Department of Electrical Engineering, Milligan University, Johnson City, TN 37682, USA; 4 School of Electronics Engineering, College of IT Engineering, Kyungpook National University, Daegu 41566, Republic of Korea

**Keywords:** atmospheric pressure plasma, multi-bore tube, phosphorus compound decomposition, plasma polymerization, plasma processing, plasma treatment

## Abstract

In this study, we describe an atmospheric pressure plasma jet (APPJ) device made of four-bore tubing operable in inhospitable humid environments and introduce two potential applications of liquid material processing: decomposition of aqueous phosphorus compounds and solution-plasma polymerization. A four-bore tube was used as the plasma transfer conduit and two diagonal bores contained metal wires. In the proposed APPJ device, the metal wires serving as electrodes are completely enclosed inside the holes of the multi-bore glass tube. This feature allows the APPJ device to operate both safely and reliably in humid environments or even underwater. Thus, we demonstrate that the proposed electrode-embedded APPJ device can effectively decompose aqueous phosphorus compounds into their phosphate form by directly processing the solution sample. As another application of the proposed APPJ device, we also present the successful synthesis of polypyrrole nanoparticles by solution plasma polymerization in liquid pyrrole.

## 1. Introduction

Nonthermal atmospheric pressure (AP) plasma is a weakly ionized gas medium with ionized charged particles, exciting species with varying energy levels, highly reactive but short-lived radicals, and free electrons [1,2,3,4,5]. Because these byproducts of nonthermal AP plasma are effective agents for various materials, plasma technology has been effectively applied in material processing over the past few decades, despite the difficulty in diagnosing whether AP plasma is in contact with materials [6,7]. The non-equilibrium discharge behavior owing to partial ionization allows to attain high electron energies in the plasma medium while retaining ions and neutral species at room temperature [8]. The presence of various radicals and highly energetic electrons at low gas temperatures indicates that nonthermal AP plasma is particularly advantageous when processing heat-sensitive materials, such as organic materials, polymers, and volatiles [9,10,11,12,13]. In addition, because there are no bulky chambers or complicated vacuum components in the plasma generation system, and no chemical waste is generated after the process, material processing with AP plasma is generally considered an eco-friendly process with the advantages of having a simple overall experimental setup, fast processing, and easy maintenance. Regarding using nonthermal AP plasma, material decomposition and synthesis, two representative material processing methods, are gradually increasing in popularity, and research on various AP plasma device designs is attracting attention as well.

The AP plasma jet (APPJ) device, which can create a nonthermal AP plasma having the simple configuration of an electrode and a conduit, ignites the discharge gas flowing through the conduit by a powered electrode and deliver the plasma plume to the outside of the conduit [14,15,16,17]. APPJ has the advantage of being able to easily generate plasma at AP using a discharge gas, such as argon (Ar) or helium that ignites the electrical discharge better than air and delivers it close to the target material. To effectively utilize APPJ for material processing, various efforts have been made to transfer the plasma and its byproducts as close to the target as possible. There are several reports on the fabrication of various types of APPJ devices using flexible plastic tubes, as well as glass, quartz, or ceramic conduits [18,19,20,21]. Nevertheless, the difficulties related to the effectiveness of APPJ in the synthesis, decomposition, or functionalization of organic materials include the precise delivery of the reactive radicals in the plasma medium to the target and the risk of unwanted electric shock around the powered electrode owing to utilizing high voltages. In particular, when processing liquid materials using this method, it is necessary to pay attention to the malfunction of APPJ due to the undesirable electrical breakdown occurring at the electrode exposed to high-humidity environments. If APPJs can be guaranteed to operate stably and safely in inhospitable humidity conditions, applications, where APPJs can be used for liquid-material processing, will be greatly expanded. Our research group has attempted to ensure that APPJs function well in humid environments without discharge failure [22,23]. In particular, the fabrication of an APPJ device using multi-bore tubing is a promising approach to exhibiting plasma generation even in an inhospitable environment; however, there was no follow-up study except for a photo image of plasma generation [23].

Therefore, in this study, we demonstrate an APPJ device with built-in electrodes inside a plasma transfer conduit to facilitate the plasma treatment of organic materials in the liquid state. To avoid operational failure in humid environments and improve the targeted delivery of plasma, a multi-bore glass tube was employed as the plasma delivery tube. The multi-bore tube has four identical holes. Two of them are for openings in the nonthermal plasma jets and the other two are for the wire electrodes. Our research group demonstrated that the proposed APPJ can safely and stably generate plasma plumes not only in an atmospheric environment but also in water, underlining its suitability for the decomposition of aqueous phosphorus compounds related to wastewater treatment and monitoring. Furthermore, this APPJ device can be applied to plasma polymerization by generating in-liquid plasma in the monomer solution.

## 2. Materials and Methods

### 2.1. Atmospheric Pressure Plasma Jet Device and Plasma Operation System

**Figure 1:**
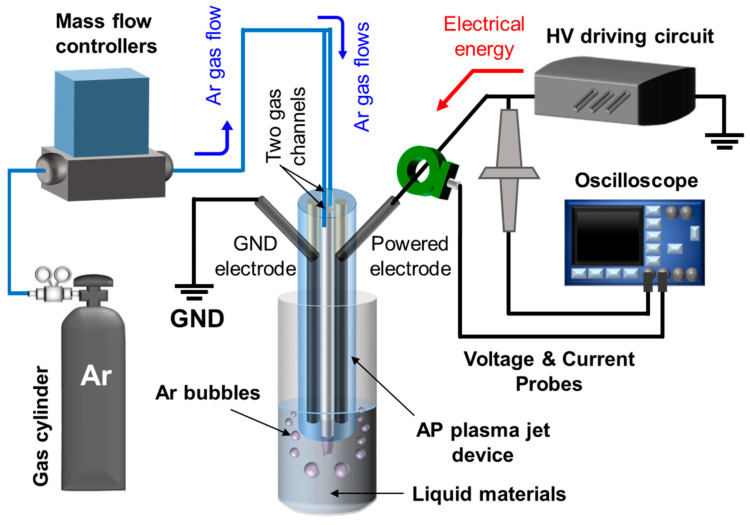
Schematic of experimental setup including an atmospheric pressure plasma jet (APPJ) device with two embedded electrodes.

The APPJ device with a four-bore glass tube and plasma generation system employed in this study was described in our previous report [23] and is shown in Figure 1. The four-bore borosilicate glass tube has four equal holes with a diameter of 1.5 mm and a total outer diameter of 6.35 mm. Two ends of the four holes were sealed with epoxy resin. Stainless steel wires with a 1.2 mm diameter were inserted into the sealed holes. The overall length of the APPJ device was 25 cm and the width was 6.35 mm, which spatially separated the resulting plasma columns and the electrical feeder by a distance of 25 cm.

A schematic of the experimental setup employed in this study is shown in Figure 1. High-purity (HP) grade Ar gas with 99.999% purity was used as the carrier gas, and a sinusoidal voltage waveform was used to power the APPJ device. A voltage probe (P6015A, Tektronix Inc., Beaverton, OR, USA) and current probe (4100, Pearson Electronics Inc., Palo Alto, CA, USA) were used along with a fiber-optic spectrometer (USB-2000+, Ocean Optics Inc., Dunedin, FL, USA) to measure the electrical and optical characteristics of the plasma plumes. The instantaneous waveforms of voltage, current and optical emission were displayed in real time on an oscilloscope (TDS3014B, Tektronix Inc., Beaverton, OR, USA).

### 2.2. Preparing a Phosphorus Compound Solution for Assessing Plasma Decomposition

A mass of 1.98 mg of β-glycerol phosphate disodium salt pentahydrate (BGP; C_3_H_7_Na_2_O_6_P·5H_2_O, Sigma-Aldrich Inc., St. Louis, MO, USA) was dissolved in 200 mL of deionized (DI) water to prepare a diluted BGP solution having a phosphorus concentration of 1.0 mg/L. The BGP solution (10 mL) was placed in a glass vial and irradiated with nonthermal plasma for up to 30 min using the proposed APPJ device. A sinusoidal voltage with a peak voltage of 6.5 kV and a frequency of 25 kHz was applied, and an Ar flow rate of 2 standard liters per minute (slm) was used to generate a nonthermal plasma to be used in the examination. The orthophosphate concentrations of the BGP samples with and without plasma treatment were measured using ion chromatography system (ICS3000, Dionex Corp., Sunnyvale, CA, USA) at the Korea Basic Science Institute (KBSI, Busan, Korea). The measurements were taken at an injection loop volume of 20 μL and column temperature of 30 °C.

### 2.3. Ascorbic Acid Reduction Method Using Phosphate Standard Solutions

1.1 mg of potassium phosphate monobasic (KH_2_PO_4_, Sigma-Aldrich Inc., St. Louis, MO, USA) was dissolved in 250 mL of DI water to prepare a phosphate standard solution having a phosphorus concentration of 1.0 mg/L. Then, DI water was further added to the standard solution to prepare phosphate standard samples containing 0.2, 0.4, 0.6, and 0.8 mg/L of phosphorus, respectively. 0.18 g of a colorimetric reagent for the ascorbic acid method (HI736, Hanna Instruments Inc., Woonsocket, RI, USA) was added to 10 mL of all standard solutions and reacted for 10 min. The absorption spectra in the visible-near infrared region of the prepared phosphate standard solutions were recorded using a spectrometer (USB-4000 UV-vis, Ocean Optics Inc., Dunedin, FL, USA). Calibration curves for the standard solutions were plotted based on the absorbance characteristics at 710 nm [24,25]. The decomposition of the BGP solution into orthophosphate (PO_4_^3−^) by plasma treatment was investigated comparatively based on these calibration curves.

### 2.4. Solution Plasma Polymerization for Examination of Plasma Synthesis

Polypyrrole (PPy) nanomaterials were synthesized from a pyrrole monomer solution using the proposed electrode-embedded APPJ device. The amount of liquid pyrrole per treatment was 25 mL, and an Ar flow rate of 500 standard cubic centimeters per minute was used. A bipolar pulse with an amplitude of 7 kV and frequency of 5 kHz was applied to the APPJ device using a high-voltage power amplifier (20/20C-HS, Trek, Inc., Lockport, NY, USA) and function generator (AFG-3102, Tektronix Inc., Beaverton, OR, USA). The pulse duration of positive and negative polarity in bipolar pulse was equal to 100 μs and the polymerization duration time was up to 6 h.

### 2.5. Preparation of Polypyrrole Nanoparticles

To separate the PPy nanoparticles synthesized from the pyrrole monomer, the processed pyrrole solution was mixed with ethanol and precipitated using a centrifuge at 13,500 rpm for 20 min. Then, the upper part of the purified solution, except for settled PPy nanoparticles, was removed using a micropipette. The settled PPy nanoparticles were added to distilled water and rinsed twice with a centrifuge under the same conditions. Finally, solid PPy nanoparticle powders were obtained after drying in an oven at 60 °C for 12 h, and then collected and fixed on copper tape.

### 2.6. Analysis and Characterization of Polypyrrole Nanoparticles

The ultraviolet-visible (UV-vis) absorption spectra of the pyrrole solution processed using the proposed APPJ were recorded over a spectral range of 250–500 nm using a UV-vis spectrophotometer (LAMBDA 950, Perkin Elmer, Inc., Waltham, MA, USA) at KBSI (Daegu, Korea).

Field-emission scanning electron microscopy (FE-SEM; SU8220, Hitachi Korea Co. Ltd., Seoul, Korea) was used to observe the shape and size of the PPy nanoparticles.

The functional groups of the nanoparticles synthesized by the plasma process were identified using Fourier-transform infrared spectroscopy (FT-IR; Vertex 70, Bruker, Ettlingen, Germany) at KBSI (Daegu, Korea). The attenuated total reflection (ATR) FT-IR spectra were measured by the average of 128 scans in the range 650–4000 cm^−1^ at a resolution of 0.6 cm^−1^.

### 2.7. Statistical Analysis

All quantitative data related to phosphorus compound decomposition are presented as mean ± standard deviation (SD). The mean and SD values were obtained from triplicate measurements of each experiment (n = 3).

## 3. Results and Discussion

### 3.1. Electrode-Embedded Atmospheric Pressure Plasma Jet Device

**Figure 2:**
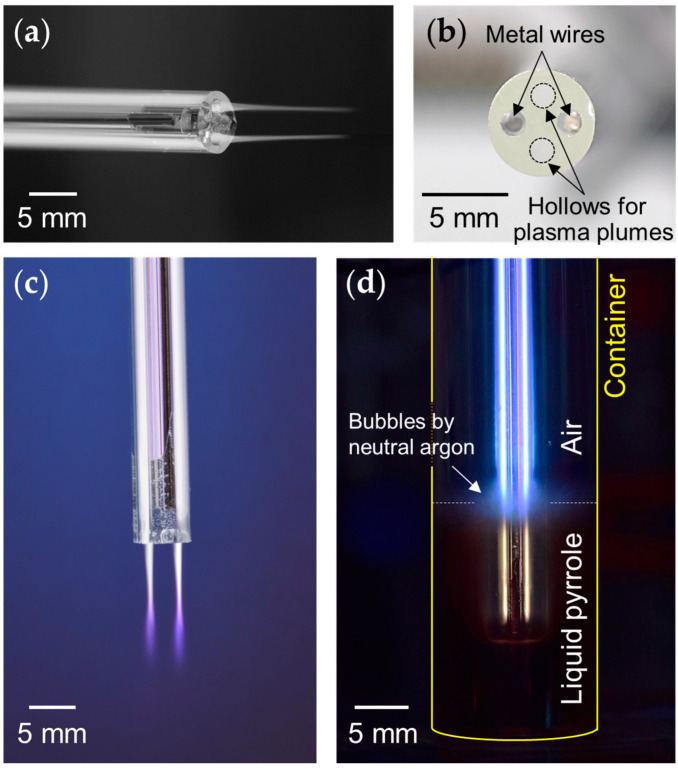
Details of the proposed APPJ device: (**a**) An APPJ device with two wire electrodes embedded in the four-bore tube. (**b**) Photograph of the four-bore glass tube with wire electrodes inserted. (**c**) Plasma plumes generated by the proposed plasma device in the air, and (**d**) plasma processing of liquid pyrrole.

As shown in Figure 2a, the two hollow ends containing wire electrodes were sealed to ensure that the device functioned properly with respect to both safety and stability. Figure 2b shows a photograph of the end of the 4-bore tube with the wire electrodes inserted. Because the diameter of this metal wire was 1.2 mm, there was only a spatial margin of 300 μm between the inner diameter of the hole and the outer diameter of the metal wire. Because of this tight spatial margin, the proposed APPJ device has the advantage that the gap between the two metal wires, the most important experimental parameter for initiating and maintaining the plasma, hardly changes regardless of the length of the plasma device. Consequently, the proposed AP plasma device can be manufactured in various lengths for processing purposes by determining the overall length of the 4-bore tube. In addition, the two-gas nozzle configuration using a 4-bore tube has the great advantage of doubling the spatial expansion of the plasma plume without any difficulty. The spatial expansion of the plasma plume can increase the plasma treatment area, resulting in faster material decomposition and polymerization processes. In the proposed APPJ device, two hollows with wire electrodes and two hollows from which plasma plumes are emitted are positioned symmetrically. Thus, creating two plasma plumes does not require twice as much power as generating one, because it still uses the same two wire electrodes. The use of two gas nozzles is an important design of the proposed APPJ device and can provide a key clue for the large-area treatment of AP plasma.

To demonstrate plasma generation in air shown in Figure 2c, an Ar gas flow rate of 1.5 slm was applied to the electrodeless hollows; subsequently, the APPJ device produced two plasma plumes of equal length of 2 cm. In this case, the operating voltage was a sinusoidal waveform with an amplitude of 5 kV and a frequency of 25 kHz. The discharge was initiated inside the 4-bore tube, through which the Ar gas flowed, and the plasma afterglow was transferred to the solution according to the gas flow. Even though the plasma plumes were observed to shorten to less than 1 mm in liquid pyrrole, the plasma device could still operate in a stable state (Figure 2d). The shorter plasma jet length in liquid than in air is due to the different densities of the media [23]. In this AP plasma device, Ar plasma is ignited inside the glass tube via two built-in electrodes, and the afterglow of the Ar plasma is emitted to the outside through two nozzles as neutral argon gas flows. When the external medium is fairly dense, such as water or liquid monomers, it is difficult for the plasma to flow out of the plasma device, resulting in very short plasma jet lengths. Therefore, using higher Ar flow rates to increase the length of the Ar plasma plume works to some extent in air, but does not work well in dense media such as liquids. However, even though the plasma jet length was reduced to less than 1 mm, numerous radicals were still present in the plasma medium and came out through the device along with the Ar bubbles.

### 3.2. Optical and Electrical Characteristics of Atmospheric Pressure Plasma Jet

**Figure 3:**
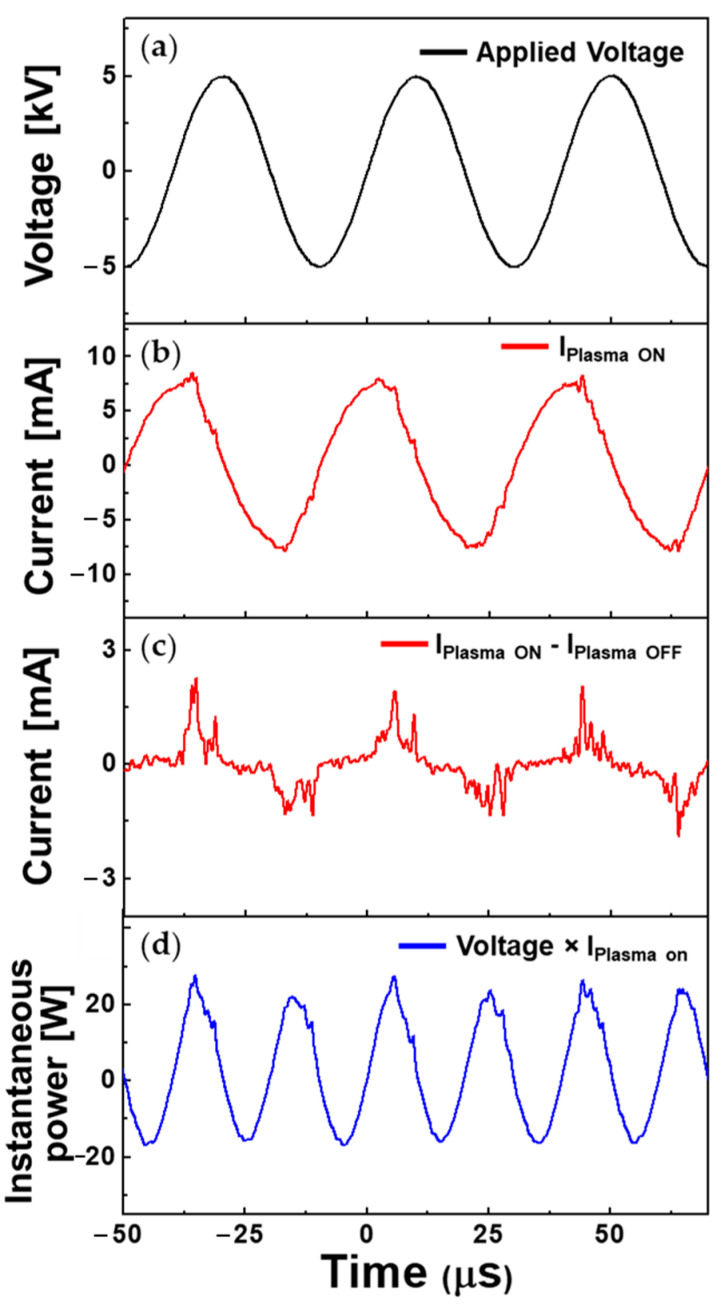
Temporal electrical behavior of plasma jet device operating in liquid pyrrole. Profiles of (**a**) driving voltage, (**b**) total current, (**c**) discharge current, and (**d**) instantaneous power as functions of time.

Compared with conventional APPJs, which only work well under normal air conditions, the proposed APPJ ensures consistent and stable operation not only under normal air conditions, but also in humid or liquid environments. The electrical characteristics measured in the proposed APPJ device operating in liquid pyrrole are depicted in Figure 3. These electrical characteristics are important because they show the general electrical properties of dielectric barrier discharges, even under liquid environmental conditions where normal APPJs cannot sustain stable driving. All measured data were averaged over 16 periods of waveforms for data reliability. Figure 3a,b presents the applied voltage and measured total current flow when alternating-current (AC) plasma is sustained. The current observed during the plasma-on state, shown in Figure 3b, consists of the discharge and displacement currents. The discharge current was acquired by subtracting the current obtained when the operating voltage was applied without discharge gas from the total current displayed on the oscilloscope when the discharge occurred. From Figure 3c, the discharge current (I_Plasma ON_–I_Plasma OFF_) appears to be periodic, according to that of the driving voltage. This periodicity indicated that the device operated in a stable state. It is also observed that the discharge current is evenly distributed in the positive and negative periods of the voltage waveforms because the two identical metal wires inserted into the 4-bore tube alternatively assume the roles of the anode and cathode during the AC discharges.

The instantaneous power consumption is shown in Figure 3d, and the average power was calculated according to the following formula:

$$P=\frac{1}{T}\int_{0}^{T}U\left(t\right)\times I\left(t\right)dt$$

where *T* is the period of the applied voltage, *U*(*t*) is the voltage signal, *I*(*t*) is the acquired current, and *t* is the time. The integrated value of the waveform of the instantaneous power during one period (0–40.0 µs) is 1.041 × 10^−4^ J. Therefore, the average power during one period is approximately 2.602 W, which is low energy consumption.

**Figure 4:**
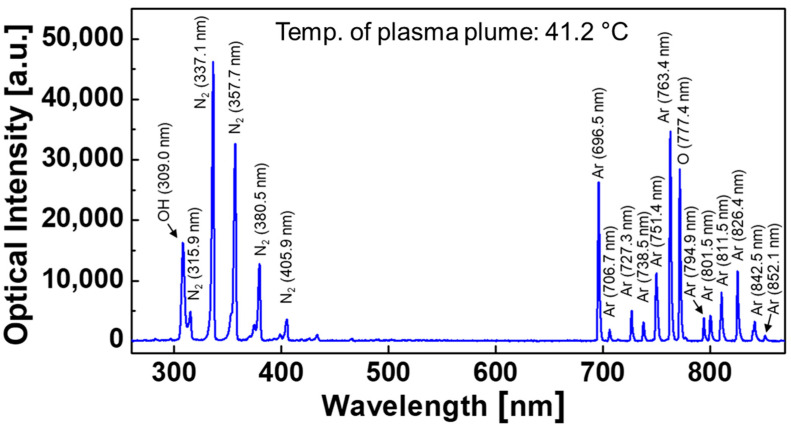
Optical emission spectra of the generated plasma plume, which was monitored using a fiber-optic spectrometer.

The optical emission spectra of the proposed APPJ were measured using a miniature spectrometer to identify the various reactive species produced by the plasma plume in the air. In this optical measurement, the temperature of the plasma plumes emitted into the air was measured to be 41.2 °C. Figure 4 shows that although Ar gas played an integral part in plasma jet production, many gaseous species, such as excited OH, N_2_, and O present in ambient air, also served as catalysts in the plasma medium. In particular, the presence of reactive oxygen and nitrogen species (ROS and RNS) is beneficial because they have been associated with the compound decomposition response.

### 3.3. Decomposition of Aquaeous Phosphorus Compounds by Atmospheric Pressure Plasma Jet

The proposed APPJ device can function safely and reliably even in harsh environments such as extremely humid environments because the ends of the two holes containing the wire electrodes are completely sealed. As shown in Figure 1, thin wire electrodes inserted into the two hollows of the four-bore tubes are completely isolated from the outside. Because the electrode has no contact with the external environment at the end of the APPJ device, two plasma jets can be maintained not only in ambient air but also in water, which greatly expands the application field of plasma processing.

Recently, several studies on the decomposition of organic/inorganic compounds using charged particles and reactive species generated by plasma have been reported [26,27,28]. Among these topics, we are interested in the decomposition of aqueous phosphorus compounds related to water quality monitoring. Because phosphorus in aquatic ecosystems exists as a variety of phosphorus compounds, the total phosphorus amount in water can be determined only when phosphorus compounds are decomposed into orthophosphate, PO_4_^3−^. The ascorbic acid method, which is widely known as the standard method for monitoring water quality, is also based on determining the amount of phosphate decomposed from diverse aqueous phosphorus compounds [29,30].

**Figure 5:**
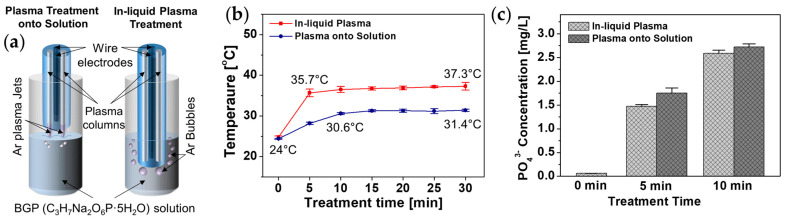
Plasma decomposition of phosphorus compounds: (**a**) Schematics of two different plasma treatments for a BGP solution by the proposed APPJ device. (**b**) Temperature change in BGP solution treated by plasma treatments. (**c**) Changes in orthophosphate concentration before and after two plasma treatments of BGP solution via ion chromatography. Data are presented as the mean ± SD of three repeated experiments.

We tested the measurement of phosphate decomposed by in-liquid plasma in a phosphorus compound solution as a potential application of the proposed APPJ device. Because the proposed APPJ device can generate nonthermal plasma not only in the air but also in water, the surface treatment of a phosphorus compound solution using plasma plumes generated in air and treatment by immersing the APPJ device in the phosphorus compound solution were investigated and compared (Figure 5a). A BGP solution containing 1.0 mg/L phosphorus was selected as an example phosphorus compound and treated with nonthermal plasma for up to 30 min using the proposed 4-bore tube-based APPJ device. Figure 5b plots the temperature changes in the BGP solution by Ar plasma jet in air and liquid Ar plasma treatments. Because the heat generated by the APPJ device is directly transferred to the solution during the in-liquid plasma treatment, the saturation temperature of the BGP solution was observed to be approximately 6 °C higher for the in-liquid plasma treatment than that for the plasma treatment on the solution surface. However, even when the BGP solution was treated with the two types of plasma for 30 min, the solution temperature was saturated to just below 40 °C, indicating that the heat generated by plasma processing did not affect the decomposition of the phosphorus compound in this experiment.

Ion chromatography measurements demonstrated that the orthophosphate concentration in the BGP solution considerably increased after 5 and 10 min of plasma exposure (Figure 5c). This indicated that the nonthermal plasma generated by the proposed AP plasma device was effective in decomposing BGP in the form of C_3_H_7_Na_2_O_6_P·5H_2_O into orthophosphate in the form of PO_4_^3−^. In particular, when the Ar plasma jet generated in the air was irradiated on the surface of the BGP solution, the amount of orthophosphate in the BGP solution was higher than that obtained when the Ar plasma jet was generated in water. This is because the nitrogen and oxygen present in the air participate in the discharge process and produce more ROS and RNS. When discharge occurs in water, ROS can be generated by water molecules, but RNS cannot be generated owing to the lack of nitrogen. Accordingly, we demonstrated that the RNS generated affected the decomposition of the BGP solution into orthophosphate and plasma the jet generated in the air was more effective in decomposing the BGP solution into orthophosphate.

However, under the condition of plasma treatment on the solution surface, it was also noticed that the plasma device should be very close to the solution target, less than 1 cm, for reliable treatment. Plasma operation using conventional APPJ generators at such high environmental humidity can create unwanted discharge at the exposed powered electrode and sometimes cause discharge failure; however, the proposed APPJ device can completely avoid this experimental risk. Moreover, in the case of the plasma treatment of the solution, because the distance between the device and the solution surface may be changed due to sample evaporation, the plasma treatment effect may change with an increase in the plasma processing time. Therefore, if a precise and reliable plasma process that does not change the plasma influence as a function of time is required, in-liquid plasma treatment is preferable.

**Figure 6:**
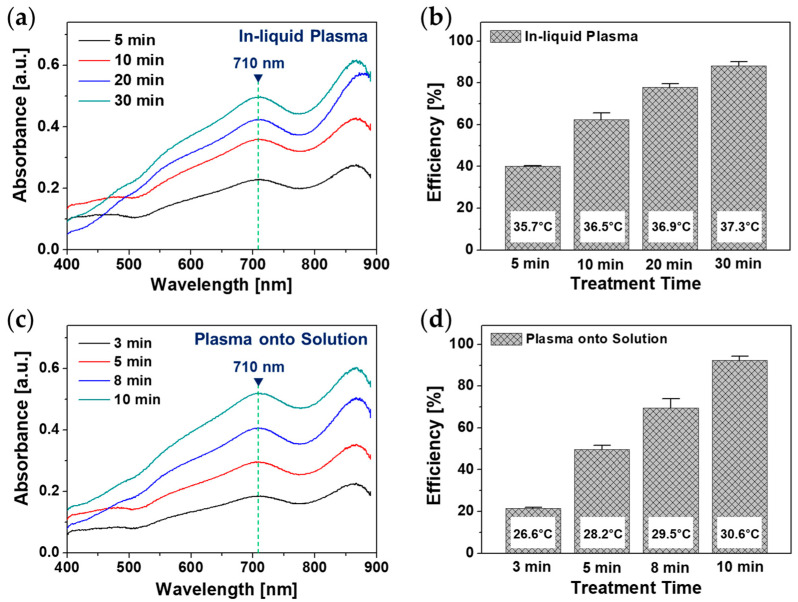
Results of ascorbic acid method: (**a**) Visible-NIR absorption spectrum curves of BGP solution treated by in-liquid plasma for 5, 10, 20, and 30 min and (**b**) efficiency of phosphorus decomposition to phosphate form via in-liquid plasma. (**c**) Visible-NIR absorption spectrum curves of Ar plasma treatment onto BGP solution for 3, 5, 8, and 10 min and (**d**) efficiency of phosphorus decomposition into phosphate form via Ar plasma treatment onto BGP solution. Phosphorus decomposition efficiencies are presented as the mean ± SD of three repeated experiments.

Figure 6a depicts the absorption spectra in the visible-near infrared region of BGP solutions treated with in-liquid plasma as a function of plasma treatment time. Based on the ascorbic acid method [31], the amount of phosphate can be quantified by comparing the absorption of the prepared phosphate standard solution at 710 nm with that of the plasma-treated samples [24,32]. Figure 6b shows the results of the decomposition efficiency of the BGP samples into orthophosphate by in-liquid plasma according to treatment time, which indicates that most of the phosphorus compounds were degraded into phosphate after in-liquid plasma treatment using the APPJ device for 30 min. Similarly, the absorption spectra and decomposition efficiency of the BGP sample with plasma treatment on its surface are shown in Figure 6c,d. In this case, observe that the processing time for most of the phosphorus compounds to be decomposed into phosphates in the plasma-treated BGP sample is shorter than 10 min. This means that the Ar plasma jet generated in the air is more effective in decomposing the BGP solution into orthophosphates than the in-liquid Ar plasma, but it is also noted that both plasma treatment approaches are sufficiently effective for decomposing BGP solutions. This result agrees well with the ion chromatography results shown in Figure 5c. The experimental results demonstrated that the plasma decomposition method available in a humid environment can be effectively combined with the ascorbic acid reduction to measure the total aqueous phosphorus concentration.

### 3.4. Plasma Polymerization in Liquid Monomer Using Electrode-Embedded Atmospheric Pressure Plasma Device

Another benefit of the proposed APPJ device is that the solution plasma process (SPP) can be applied without any operational difficulty. An SPP mainly uses a spark discharge generated in a liquid by applying an electrical impulse or high voltage between two pin-shaped electrodes facing each other with a small gap [33,34,35]. The liquid near the metal electrode is locally vaporized by Joule heating to generate a strong spark discharge, and radicals generated from liquid molecules react with each other to form nanoparticles and cool rapidly in the liquid. Because of this nanoparticle formation mechanism by solution plasma, it is difficult to synthesize organic nanoparticles that require low-temperature plasma. Our research group introduced a gas channel and dielectric barrier to synthesize polymeric nanomaterials using SPP in a previous study [36]. The use of an Ar bubble channel can significantly reduce the operating voltage while stably generating a streamer discharge, and the dielectric barrier effectively controls the discharge current.

**Figure 7:**
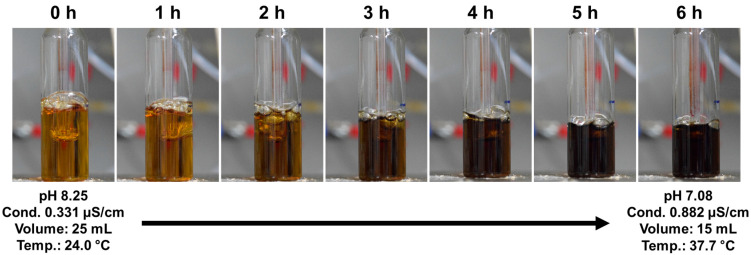
Color change in liquid pyrrole with various process times during solution plasma process (SPP) using the proposed APPJ device.

The proposed electrode-embedded APPJ device using Ar gas channels can easily generate low-temperature discharge, even in a solution. Accordingly, the proposed APPJ device was applied to PPy synthesis through SPP. Figure 7 shows the solution color change in liquid pyrrole during SPP with various processing times. The color of the liquid pyrrole gradually changed from yellowish brown to dark brown, representing that particles were formed from the liquid pyrrole. When liquid pyrrole with the initial temperature of 24 °C was treated with SPP using the proposed APPJ, the temperature of the liquid pyrrole was saturated within 30 min and maintained at approximately 38 °C until 6 h of SPP. This low saturation temperature implies that the heating of the liquid phase by SPP does not contribute to nanoparticle synthesis. Because the Ar plasma jet was generated during immersion in liquid pyrrole, evaporation and liquid disturbance by Ar bubbling inevitably occurred, which could affect the solution plasma process. Liquid pyrrole (25 mL of liquid pyrrole was evaporated by Ar bubbling, leaving only 15 mL after 6 h of SPP, and the liquid pyrrole sample was well stirred by Ar bubbling during the SPP. The pH and conductivity of the original pyrrole solution were 8.25 and 0.331 μS/cm, respectively. After 6 h of SPP, the pH was changed to 7.08 and conductivity to 0.882 μS/cm.

Oxidation of pyrrole monomers is generally known to produce chemically active pyrrole cations [37,38]. Pyrrole cations created this way polymerize with pyrrole monomers or other cations; consequently, H^+^ ions are released from the pyrrole monomer [37,38]. SPP using the proposed APPJ induced the oxidation of pyrrole, leading to the formation of pyrrole cations. The generated pyrrole cations chemically reacted with other neutral pyrrole molecules or pyrrole cations during SPP to first form pyrrole oligomers and finally form PPy particles in liquid pyrrole. Based on the conductivity and pH data of the SP-treated liquid pyrrole (Figure 7), it can be inferred that pyrrole ions, particularly cations, were formed during the SPP.

**Figure 8:**
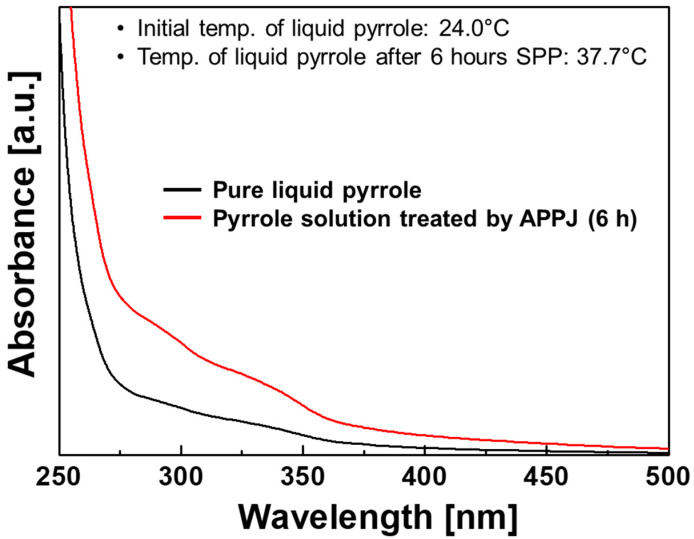
Ultraviolet-visible (UV-vis) spectra of the pure liquid pyrrole and pyrrole solution treated by the proposed APPJ device for 6 h.

Figure 8 shows UV-vis spectra of pure liquid pyrrole and pyrrole solution treated with the proposed APPJ for 6 h. The pyrrole samples with and without plasma treatment exhibit significant differences in absorbance over the 275–400 nm region, owing to the presence of pyrrole oligomers in the plasma-treated pyrrole sample. In particular, absorptions near 275 and 320 nm indicate the presence of bipyrrole and terpyrrole, respectively [39]. Thus, the UV-vis results demonstrate that pyrrole monomers were synthesized in larger units by SPP using the proposed APPJ. In addition, it is generally known that the pH of the solution decreases during PPy synthesis owing to liberated H^+^ ions [40]. Therefore, the experimental results depicted in Figure 7 and Figure 8 indicate that PPy is synthesized in the liquid pyrrole by SPP using the proposed APPJ device.

**Figure 9:**
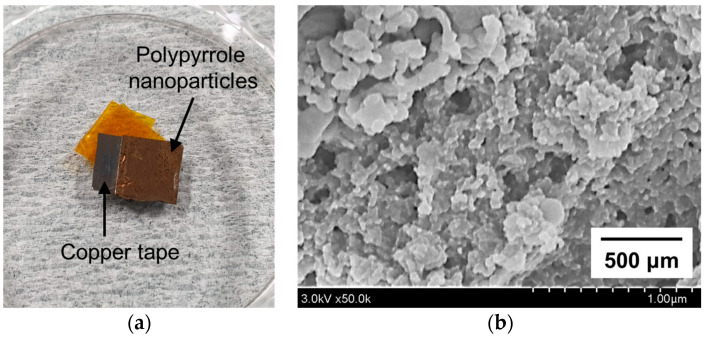
Sample of polypyrrole (PPy) nanoparticles: (**a**) Photograph of PPy nanoparticles collected on copper tape for FE-SEM measurements and (**b**) FE-SEM image of PPy nanoparticles synthesized by the proposed APPJ device.

**Figure 10:**
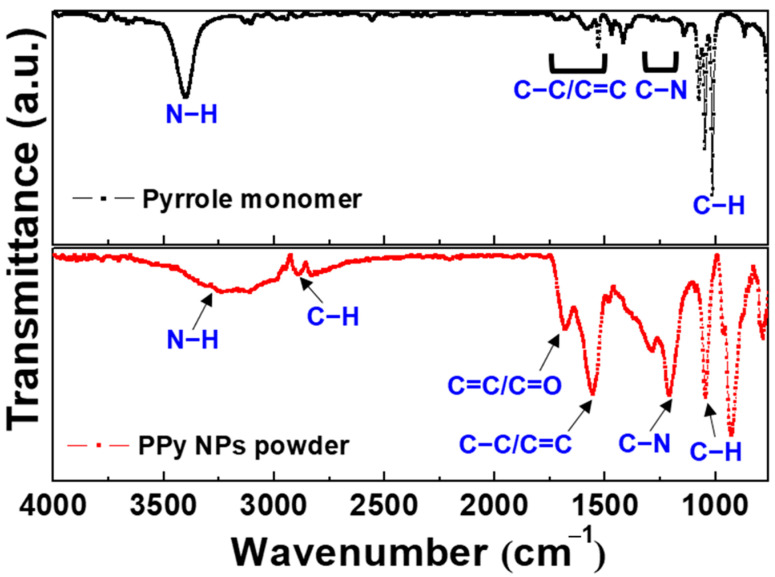
Fourier transformation infrared spectroscopy (FT-IR) spectra of pure pyrrole monomer (upper graph) and PPy nanoparticles synthesized by SPP using the proposed APPJ device (lower graph).

Figure 9a shows a sample of PPy nanoparticles synthesized for 6 h and then collected on copper tape for morphological analysis using FE-SEM images. As depicted in the FE-SEM image in Figure 9b, the synthesized nanoparticles exhibited a small spherical-like structure with a size distribution of approximately tens of nanometers to 250 nm. Figure 10 plots the FT-IR spectra of the pyrrole monomer (upper graph) and PPy nanoparticles (lower graph) synthesized by SPP using the proposed APPJ device. In the FT−IR spectrum of the pyrrole monomer, the peaks in the 3500–3300 and 1150−1000 cm^−1^ ranges originated from the stretching of the N−H bond and in-plane bending of the C-H bond, respectively. The aromatic ring stretching in the C=C/C−C and C−N bonds were observed as peaks in the 1750−1500 and 1350−1200 cm^−1^ ranges, respectively [41]. In the FT−IR spectrum of PPy nanoparticles, the band around 3300 cm^−1^ is attributed to the stretching vibration of the N−H bonds. The peaks at 2889 and 1045 cm^−1^ are attributed to the stretching and in-plane bending of the C−H bonds, respectively. The peak at 1681 cm^−1^ can be attributed to C=C/C=O stretching. C−C/C=C and C−N stretching in the pyrrole ring have been observed as the peaks at 1556 and 1211 cm^−1^, respectively. From the FE-SEM and FT-IR results, it can be concluded that PPy nanoparticles were successfully synthesized under low-temperature plasma.

## 4. Conclusions

In summary, in this study, we proposed an electrode-embedded APPJ device made of 4-bore glass tubing for use in humid environments. The device configuration, namely, having electrodes isolated from the environment and spatial separation of the plasma jet and electrical input, allows stable and safe operation in a humid environment or even in water. The proposed APPJ device has Ar gas bubble channels and a dielectric barrier inside the plasma delivery conduit; therefore, it can stably generate low-temperature plasma regardless of the external environment. We demonstrated that aqueous phosphorus compounds can be effectively decomposed into phosphate forms by plasma treatment using the proposed APPJ device. Preliminary studies on the decomposition of phosphorus compounds reported that the decomposition process using nonthermal plasma could be used as a new alternative to the pretreatment of the ascorbic acid method to verify the total phosphorus content in freshwater. As another potential application of the proposed APPJ device, PPy nanoparticles were synthesized in liquid pyrrole via solution plasma polymerization. By applying the proposed APPJ device to synthesize PPy nanoparticles using SPP, thermal damage such as pyrrole carbonization and electrode erosion can be avoided. SPP using the electrode-embedded APPJ device is expected to improve the quality, purity, and uniformity of the resulting organic nanomaterials. In addition, the proposed APPJ has a low energy consumption during operation, which is reasonable enough to transfer this technology from the laboratory to the industry. An excellent approach to effectively increase the plasma processing/processing capacity for transferring this technology to the industry is to consider using multiple plasma devices as an array.

## Data Availability

Not applicable.
